# KIT performed as a driver gene candidate affecting the survival status of patients with stomach adenocarcinoma

**DOI:** 10.18632/oncotarget.19598

**Published:** 2017-07-26

**Authors:** Shengli Pan, Jin Tan, Yingying Deng, Ben-Hai Wan, Xiao-Yu Zhang, Bu-Gao Guan

**Affiliations:** ^1^ Shanghai Eighth People’s Hospital, Shanghai, China; ^2^ Department of Thoracic Surgery, Huai’an First People’s Hospital, Nanjing Medical University, Huai’an, China; ^3^ Department of General Surgery, People’s Hospital of Jinhu, Huai’an, China; ^4^ Division of Gastrointestinal Surgery, Department of General Surgery, The Affiliated Huai'an Hospital of Xuzhou Medical University and The Second People’s Hospital of Huai’an, Huai’an, China

**Keywords:** stomach adenocarcinoma, prognosis, TCGA, KIT, driver gene candidates

## Abstract

Stomach adenocarcinoma is estimated to cause 10,000 deaths in the US in 2016 and is the third most deadly cancer in China. We aim to identify the proteins and the genes that have impact on the prognosis of patients with stomach adenocarcinoma. Data of patients with stomach adenocarcinoma were retrieved from The Cancer Genome Atlas (TCGA). Proteins whose expression levels were highly correlated with survival status of patients were figured out. The expression levels of their mRNAs and their roles in the pathway were used to determine the driver gene candidates. The effects of mutations on the genes encoding KIT on mRNA expressions were carried. Ten antibodies were figured out to have significant correlation with stomach cancer prognosis. The coefficients of COXPH models matches their roles in the previous studies. The expression levels of mRNAs versus proteins suggested that KIT might act as a driver gene, which was also the central in the pathway of other selected proteins. The missense mutations on the gene encoding KIT led to the low expression of its mRNAs and there were much fewer nonsense mutations compared with other genes. It suggested that the important role of KIT as an oncogene in the progression of cancer, as well as a tyrosine-protein kinase during the normal activity. Ten antibodies, corresponding to fifteen proteins, were highly correlated with patients’ survival time, within which KIT played a critical roles. It suggested that KIT might be used as biomarker or as target of cancer therapies.

## INTRODUCTION

It was estimated that there would be over 26,000 new stomach cancer cases and over 10,000 deaths caused by it in the United States in 2016 [1]. In China, stomach adenocarcinoma is the third most prevalent cancer, as well as the third most deadly [2]. The 5-Year relative survival rate of stomach adenocarcinoma increased from 20% before 1990 to 30% at present compared with any average survival rate of 69% for all kinds of cancer [1]. It prevalence and mortality urged us to find out the factors affecting its incidence and prognosis.

The direct molecules regulating the body status are proteins instead of DNA or RNA. However, most of cancer studies at present focused on mRNA expression levels, mutations or even miRNA expression levels, but few studies used large-scale protein expression levels considering the difficulties to obtain such information. Luckily, M.D. Anderson Reverse Phase Protein Array Core provided the expression levels proteins which are targeted by 218 different antibodies on TCGA. Such data can help us to study the cancer further. On the meantime, there are various differences, from mutations to mRNA expression levels, from copy number variation to protein translation, between cancer patients and normal populations. However, most of differences were passengers which were neutral or influenced by other aberrant behaviors. Driver genes, which are responsible for cancer development through specific alterations, were hard to be figured out unfortunately [3].

In this study, we started from protein expression levels to find out the proteins that were significantly correlated with survival time of patients. Afterward, mRNA expression levels and somatic mutations information will be utilized to identify the driver gene candidates. The final results suggested that KIT, as well as the genes encoding these proteins, might play a critical and central role in the survival status of stomach patients.

## RESULTS

### Proteins correlated with survival status

**Figure 1:**
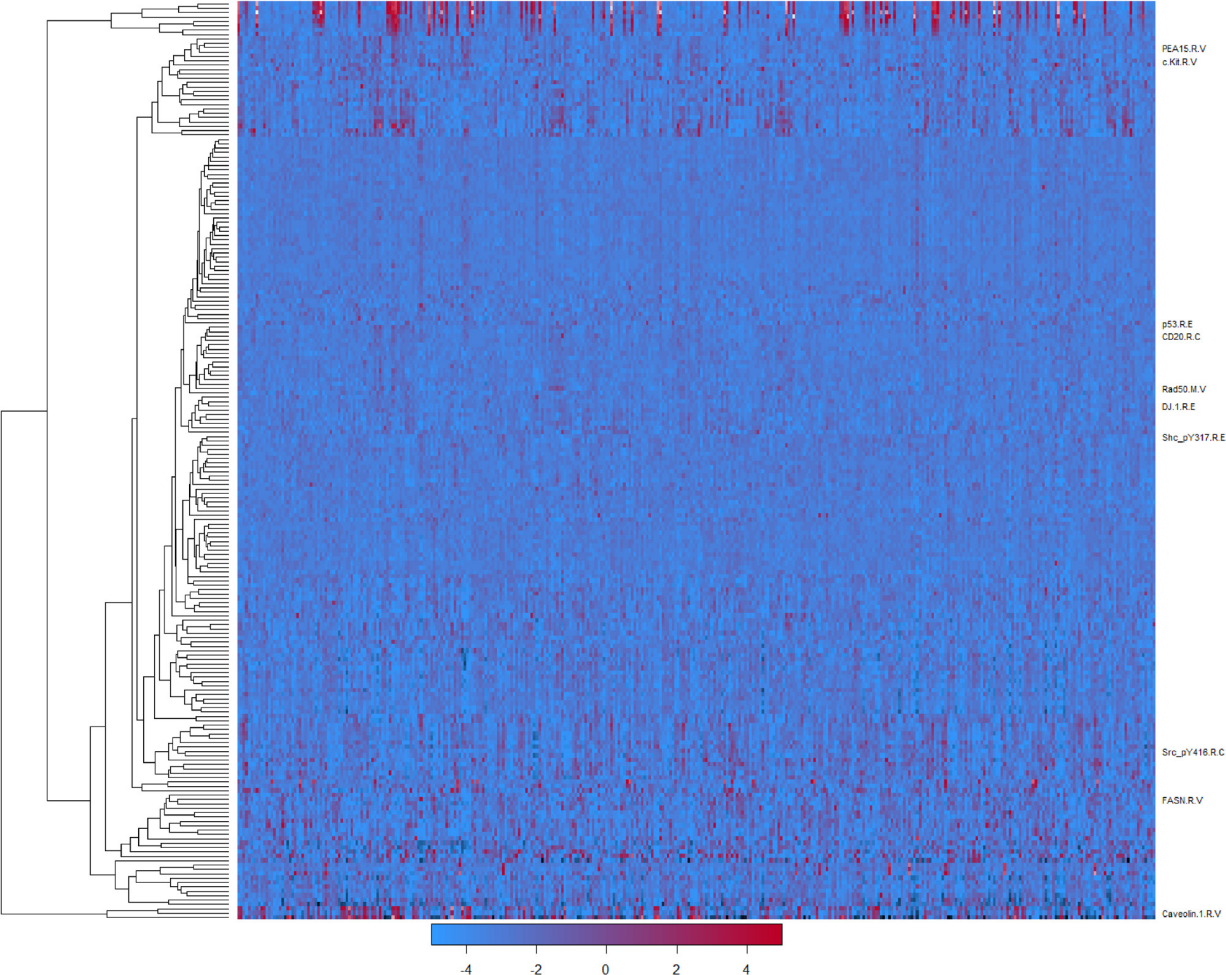
Heatmap for the expression levels of proteins The rows represented different antibodies and the columns were patients. Red meant the high expression levels and blue meant the opposite. The antibodies having a p-value lower than 0.05 in the COXPH model were annotated on the right. Antibodies that were unavailable in any patients were omitted.

There were totally 218 antibodies used to detect protein expression levels, though some antibodies unavailable for a certain group of patients. The overall expression patterns for these antibodies were plotted as a heatmap (Figure 1).

COXPH models were built separately for all the antibodies based on 347 patients with protein expressed information. Ten antibodies with a p-value less than 0.05 were extracted. The detailed information for these antibodies was listed in Table 1. Campared with previous studies, we found that most of these genes were tumor suppressors or activators. Their role also corresponded to their coefficients of the COXPH model, except for Src_pY416. Interestingly, Src_pY416 antibody targets six different proteins, which might make it more complex to figure out theirroles on the survival status. We could not determine which protein(s) resulted in the significant results with limited data.

**Table 1:** The summary of antibodies that highly correlated with survival status of patients with stomach cancer

Antibody	Protein	coef	exp(coef)	p_value	Note
Shc_pY317	SHC1	-1.24	0.29	0.041	Tumor suppressor
p53	TP53	-1.08	0.34	0.017	Tumor suppressor
Src_pY416	SRC, LYN, FYN, LCK, YES1, HCK	-0.69	0.50	0.024	Proto-oncogene
FASN	FASN	-0.56	0.57	0.031	
Caveolin-1	CAV1	0.38	1.46	0.013	Tumor suppressor
c-Kit	KIT	0.69	2.00	0.026	Tumor activator
DJ1	PARK7	1.10	3.01	0.030	Tumor activator
PEA-15	PEA15	1.23	3.44	0.012	Tumor activator
Rad50	RAD50	1.62	5.03	0.001	Tumor activator
CD20	CD20	2.23	9.35	0.031	Tumor activator

For these 10 antibodies, their expression patterns were not totally the same (Figure 1), though all of them were highly correlated with survival time of patients. This suggested their different roles in the stomach cancer progress.

**Figure 2:**
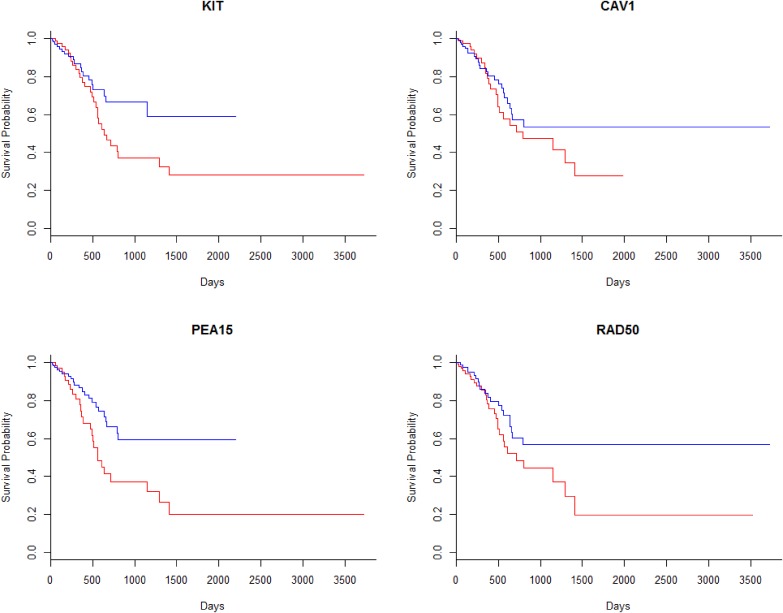
The survival curves of the patients based on the expression levels of the specific proteins Patients were separately into two even groups according to the hazard rate. The blue lines represented the patients with low hazard rate and the red lines represented high risk.

The Kaplan-Meier survival curves of four proteins were plotted as examples in Figure 2. It indicated that some of these proteins were able to separate patients with good prognosis and poor prognosis, though some of them, like CAV1, failed in separating the two groups.

### Networks and pathways of survival related proteins

**Figure 3:**
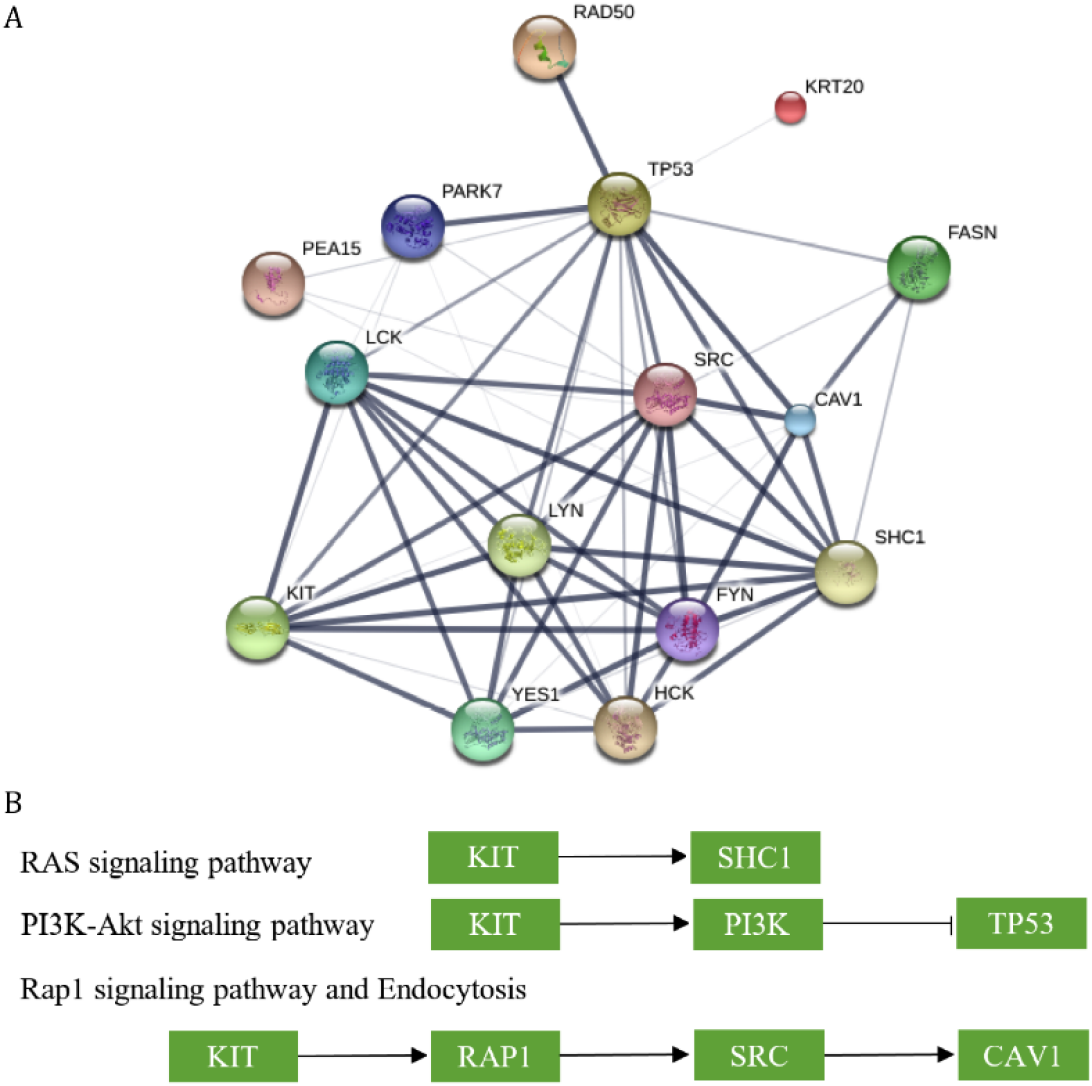
The networks and pathways involvig proteins that were correlated with patients’ survival time **(A)** The protein-protein interactions between proteins. The confidences of the interactions were annotated by the width of edges. **(B)** The selected regulated relationship between KIT and other proteins.

These proteins were highly correlated with each other. Most of these proteins had intact protein-protein interactions with high confidence and all of them had some certain evidences to suggest that they had physical interactions (Figure 3A). Such results indicated that any aberrant behavior of one protein might affect all of other proteins.

The proteins interacted with each other and most of proteins acted as a hub, including KIT, proteins targeted by Src_pY416, TP53 and so forth. It was difficult to figure out the most important proteins in such network, but when we looked at the pathway they involved, KIT performed as a central part among these proteins. When searching for the pathways involving these proteins on KEGG, it seemed that KIT was the upstream of most of other proteins (Figure 3B). For example, in the RAS signaling pathway, KIT directly activates SHC through protein-protein interaction; KIT is also the most upstream protein in PI3K-Akt and is able to regulate the function of TP53 indirectly; it works with other proteins to convert inactive RAP1 into active status and in further active SRC and CAV1. All of the pathways shared with KIT were listed in Table 2. The pathway information is unavailable or incomplete for some genes, whichwere not included in the table.

**Table 2:** The shared pathways with KIT

Pathway ID	KIT involved pathway	Shared proteins
hsa04014	Ras signaling pathway	SHC1
hsa04015	Rap1 signaling pathway	SRC
hsa04072	Phospholipase D signaling pathway	SHC1, FYN
hsa04144	Endocytosis	CAV1, SRC
hsa04151	PI3K-Akt signaling pathway	TP53

### Driver proteins versus passengers

Driver genes for cancer were responsible for the cancer incidence, progression and prognosis. Even though the levels of protein mentioned above seemed highly correlated with patients’ survival time, it is difficult to say that these proteins and the genes encoding them caused the phenomenon directly. After all, the levels of proteins were regulated by complex microenvironment including the different ion concentration, other proteins, miRNAs, mRNAs and so forth.

**Figure 4:**
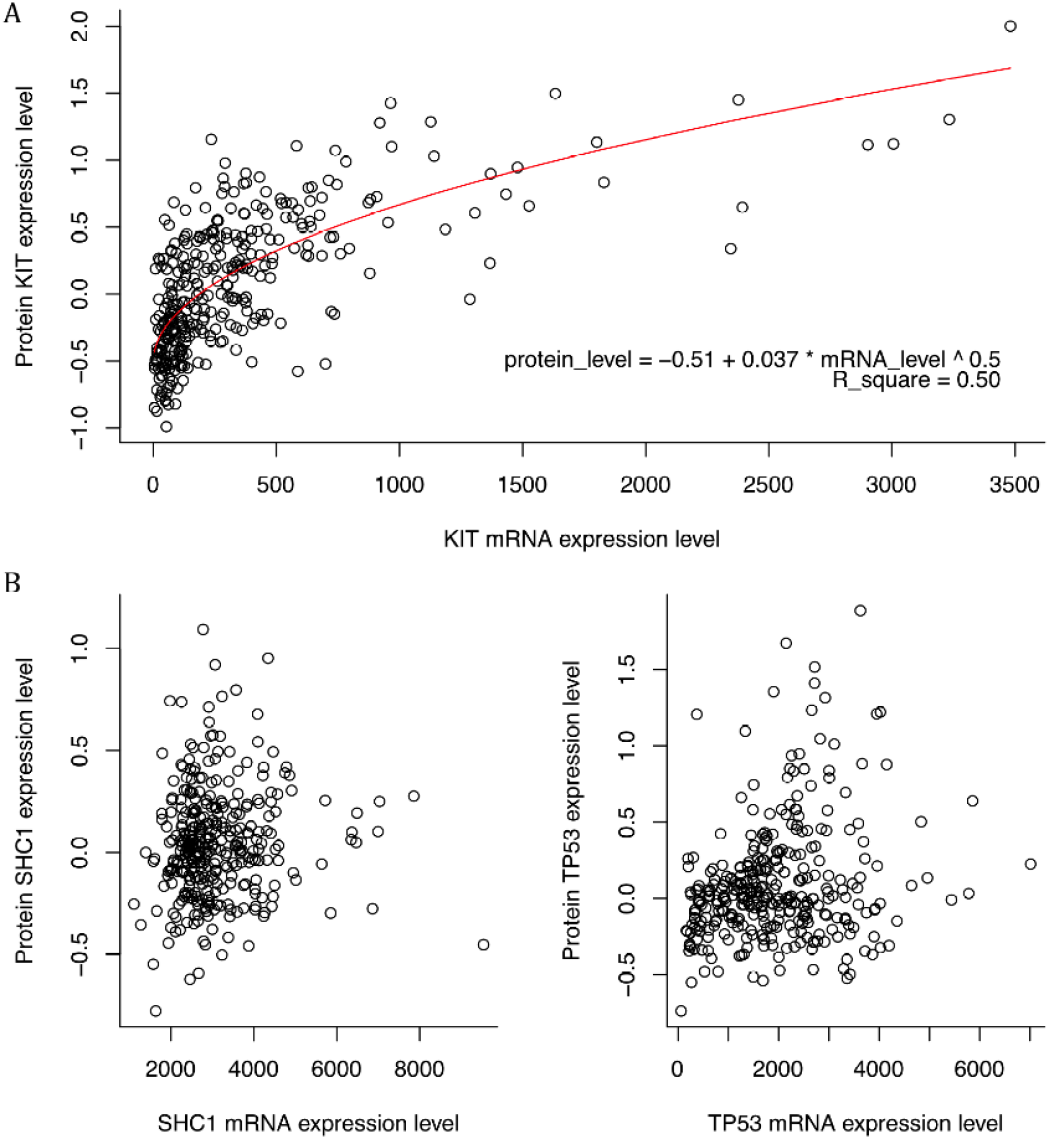
The scatter plot of mRNA expression levels versus corresponding protein expression levels **(A)** The plot of KIT. The curve of regression was plot as red which had a R-squared as 0.5. **(B)** The plot of SHC1 and TP53. Except for KIT, none of survival-related proteins had significantly correlation with their mRNA expression levels. Only two examples were shown here.

It was reported that only one-thirds of mRNA expression levels were positively related with their protein expression levels with high correlation coefficients [4]. If the mRNA expression levels were significantly correlated with the protein expression, it became a good support that the impact of protein on the patients’ survival time might come from the mRNA expression level and in future the DNA methylation and SNPs instead of regulation of other factors. Here only KIT had a significant correlation between mRNA expression levels and protein expression levels with a p-value lower than 2.2e-16 (Figure 4A). Other proteins had a low correlation coefficients and Figure 3B showed two examples The correlation coefficient for KIT was higher than 0.50, higher than the cutoff used in study of Gry, Rimini [4], which suggested that the relation is robust. Other proteins had poorer correlation.

It seemed that KIT played a central role in the analysis of pathways. And the correlation between mRNA expression levels and protein expression levels also suggested that KIT might be a driven genes instead of passenger genes. Also, the poor correlation of other proteins and the tight relation with KIT provided a possibility that KIT regulated the levels of other survival-related proteins and worked together to affect the prognosis of stomach cancer.

We also used the mRNA expression levels of these genes to fit the COXPH model but it seemed that they were not good biomarkers for the survival time. Even for the mRNA of KIT, the p-value was close to 0.1.

### Mutations of gene encoding KIT

We have suggested that the level of KIT mRNA had direct impact on its protein expression levels, and afterwards we would like to study the relationship between the DNA SNPs and mRNA expression levels.

**Figure 5:**
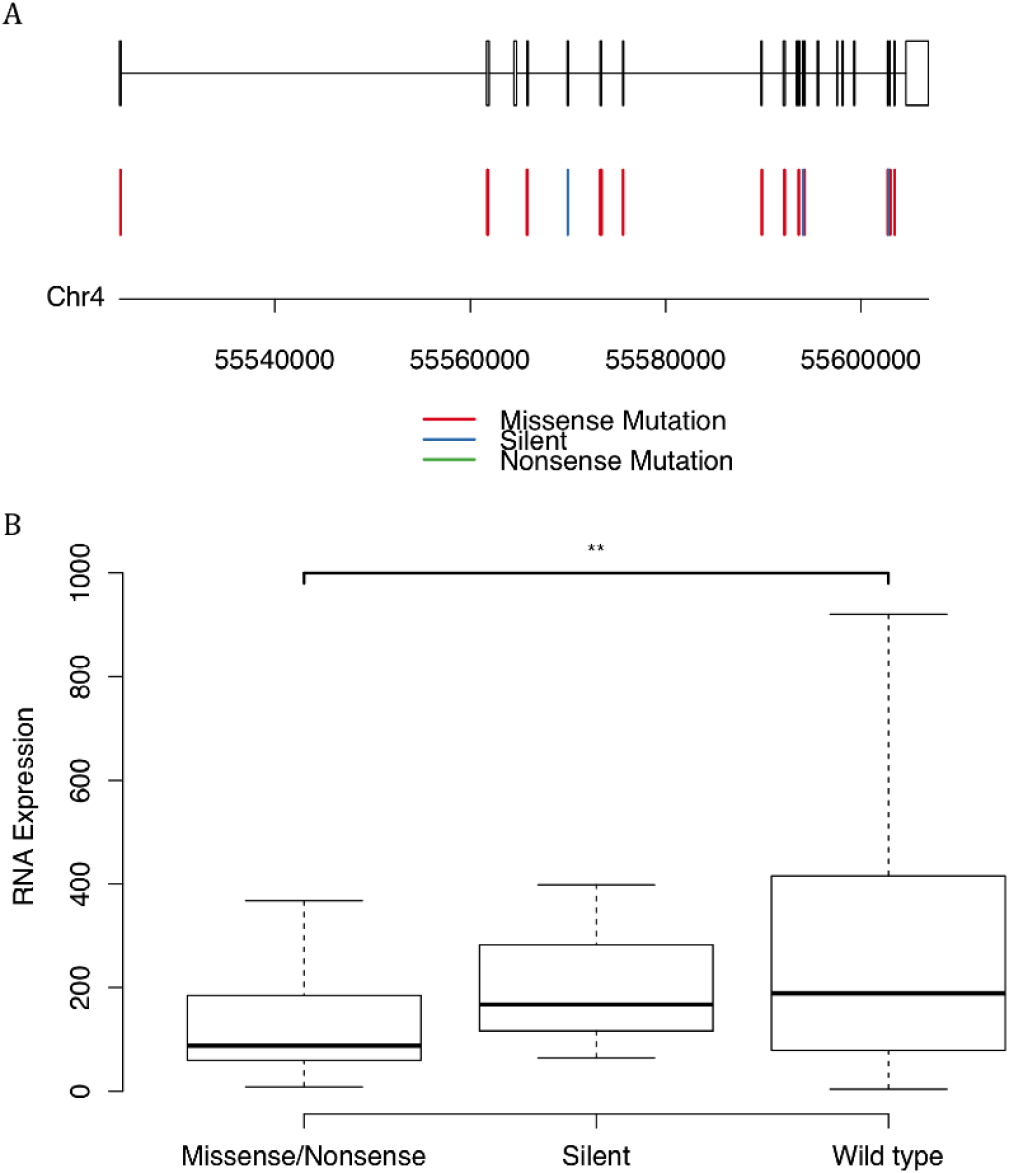
Mutations of the gene encoding protein KIT **(A)** The mutation positions and types. **(B)** The mRNA expression levels versus the status of mutations. The symbol ‘**’ represented a p-value lower than 0.05.

First, most of the mutations on KIT were missense mutations and silent mutations (Figure 5A), unlike other genes which had a lot of nonsense mutations. Second, patients with missense mutations on KIT had significantly lower level of mRNA with a p-value of 0.016 than patients without mutations (Figure 5B). Even for the patients with only silent mutaions, their mRNA levels seemed a little lower than wild-type, though the p-value is insignificant probably caused by the small sample. This hinted that the mutations on KIT might be able to affect the stability or transcription of its mRNA.

**Figure 6:**
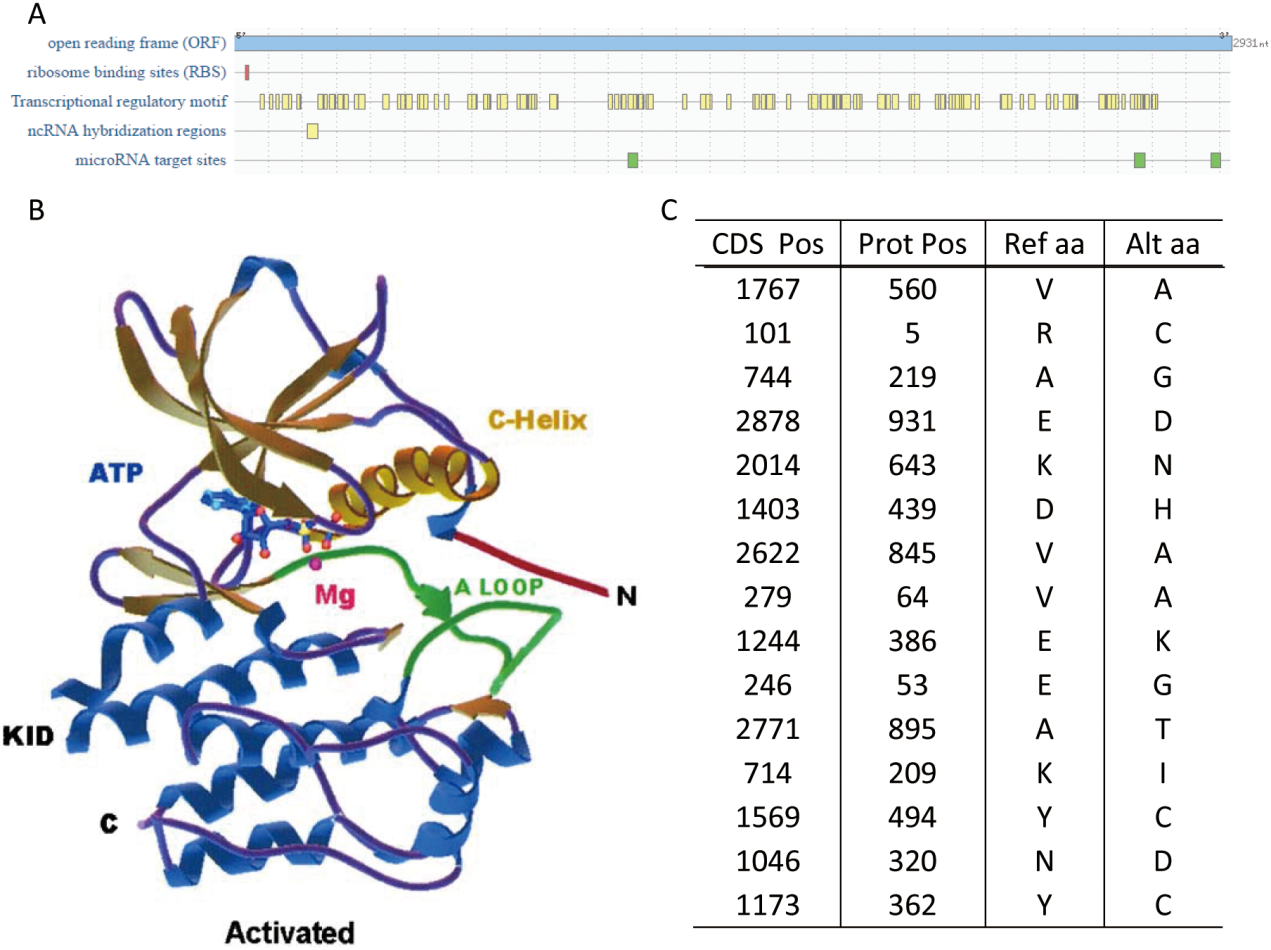
Genomic mutations of KIT **(A)** The cis-regulatory elements on KIT mRNA. **(B)** The protein structure of KIT. This graph came from the paper of Roskoski [5]. **(C)** The missense mutations in the patients from TCGA.

The overall mutations were summarized in Figure 6C. To find out the influence of these mutated sites, the cis-regulatory elements on the mRNA were predicted on RegRNA 2.0 (Figure 6A) and the protein structure was predicted using SWISS-MODEL (figure not shown). Due to the size of the protein, the SWISS-MODEL only simulated part of the protein and the whole protein structure reported by Roskoski [5] was shown in Figure 6B.

## DISCUSSION

### Missense mutations on KIT

KIT is a tyrosine-protein kinase, which performed various functions. It played important role in the process of hematopoiesis, melanogenesis, and gametogenesis [6]. It also regulates cell shape, motility, and adhesion via cytoskeletal changes. KIT is expressed as a glycosylated transmembrane protein with an extracellular domain, a transmembrane region, and a tyrosine kinase domain (Figure 6B). As a result, any nonsense mutations might be lethal and any missense mutations affected the body dramatically. It was reported that the allelic variants of KIT would lead to various diseases including piebaldism [7], mast cell leukemia [8], gastrointestinal stromal tumor [9] and so on.

The missense mutations on genes encoding KIT were summarized in Figure 6C. One intriguing finding is that most of these mutations were not known oncogenic mutations, except for the protein position 560, whose mutations will lead to gastrointestinal stromal tumors and mastocytosis [5]. Also, considering the mRNA expression levels in patients with KIT mutations and those without mutations, these missense mutations may play a more important role on its mRNAs. KIT mRNAs owned various cis-regulatory elements (Figure 6A) with functions to stabilize, mobilize and translate KIT.

Another important feature of the mutations on genes encoding KIT is that most of the mutations were missense mutations or silent mutations. We also checked the mutation status of other oncogenes and their mutations were most nonsense mutations. This may suggest the critical function of KIT *in vivo* except for its contribution for the cancer progression. However, the bench experiments, which engineer the KIT gene by inducing mutations, knockout or duplicating, are needed in further to validate our assumptions.

### Utilization of protein expression levels

We can easily find various cancer related papers talking about mRNA expression levels, but much fewer papers paid the attention on the protein expression levels. The most important reason is that it is quite hard and expensive to obtain the protein expression level. Here, we suggested that utilizing public data from TCGA and other database was a good choice to analyze protein information since these protein information cannot be found in the TCGA data matrix. Also, starting from the protein expression levels, we can directly find the molecules affected our body in a direct way. On the other hand, the protein expression levels were measured through antibody, which could not distinguish similar proteins. For example, antibody Src_pY416 targeted SRC, LYN, FYN, LCK, YES1 and HCK. In this study, we simply assumed that all the targets of Src_pY416 were associated with the patients’ survival, though more experiments were needed to identify the exact associated proteins.

## MATERIALS AND METHODS

### TCGA stomach cancer

Clinical information, protein expression levels, mRNA expression levels and somatic mutation information of patients with stomach adenocarcinoma were retrieved from The Cancer Genome Atlas (TCGA). The protein expression levels came from the platform of M.D. Anderson reverse phase protein array. The expression level of mRNA and somatic mutations were obtained under platform of Illumina HiSeq 2000 RNA sequencing version 2 and Illumina DNA sequencing, respectively. This dataset contains 478 patients, within which 347, 443 and 439 patients had protein, mRNA and mutations information.

### Survival related proteins

There were totally 218 antibodies used to detect protein expression levels. With 347 patients having protein expressed information, COXPH models were built separately for all the antibodies [11]. Ten antibodies with a p-value less than 0.05 were extracted. The Kaplan-Meier survival curves [12] were plotted to visualize the performance by dividing patients into two even groups using hazard rate from COXPH model.

### Network and pathways

The protein-protein interactions among these proteins were plotted using STRING [13]. Every proteins were searched in the database KEGG to find out the pathway they involved and the regulation relationship among them [14].

### Identification of driven protein

The expression levels of mRNA corresponding to the proteins related with survival status of patients with stomach adenocarcinoma were extracted. The relationship between mRNA expression levels versus protein expression levels were shown as scatterplots. Regression was then carried out. KIT was found to have high correlation between mRNA and protein expression levels. The following analysis would focus on this protein and the gene encoding it.

### Analysis on KIT

Mutations of the genes encoding these proteins were available in the platform using Illumina DNA sequencing. The sequences of DNA and also the gene model were retrieved from USCS [15]. The differences of mRNA expression levels between patients with or without KIT mutations were performed using Student’s T test and visualized using box-and-whisker plots.

The protein structure of KIT was predicted using SWISS-MODEL [16] and the cis-regulatory elements on the KIT mRNA were analyzed through RegRNA 2.0 [17].

## CONCLUSIONS

In this study, we at first found the proteins highly correlated with survival time of patients with stomach adenocarcinoma. Based on this analysis, KIT was suggested to be a critical and central molecule in the biological process and it performed as a driver gene instead of passenger genes. As a result, it may perform in an aberrant way in early stage that it can be used as a good biomarker. Also the targeted drugs on KIT may be the most critical. There are already targeted therapies targeting KIT, including Axitinib, Cabozantinib, Imatinib, Pazopanib, Sorafenib and so forth [10]. More targeted therapies working on KIT may be found in the future.
